# OPTC-1. Proteomic analysis of genetically stratified low-grade meningioma

**DOI:** 10.1093/noajnl/vdab070.022

**Published:** 2021-07-05

**Authors:** Yeasmin Akther, Claire Adams, Vikram Sharma, Claudia Barros, Matthew Banton, Oliver Hanemann

**Affiliations:** University of Plymouth, Plymouth, UK

## Abstract

**Introduction:**

Meningioma are the most common primary intracranial tumour. According to WHO, ~80% tumours are benign grade I. Although, some grade I tumour clinically show aggressive behaviour. Radio-surgery are the main therapeutic approaches, chemotherapies are ineffective. Accurate biomarkers for clinical management are lacking. The mutational profile of low-grade meningioma is well-defined, with non-*NF2* mutated tumours harbouring recurrent mutations in genes including *TRAF7*, *KLF4*, *AKT1* and *SMO*. Here, we aim to identify novel biomarkers and therapeutic targets of genetically stratified low-grade meningioma by characterising the proteomic landscape.

**Materials and methods:**

Meningioma specimens were stratified according to mutational background: *AKT1*^*E17K*^*/TRAF7*, *KLF4K409Q/TRAF7* and *NF2*^*-/*-^. Proteins were separated by SDS-PAGE followed by in-gel tryptic digestion and sample preparation for LC-MS/MS analysis. Raw mass spectrometry data files were processed by MaxQuant and Perseus software. Quantitative phospho-proteomics was performed using TMT-10plex labelling approach followed by motif analysis using motif-X algorithm. GO enrichment analyses were performed using DAVID against all human proteins.

**Results and Conclusions:**

We have quantified 4162 proteins across all mutational meningioma subgroups and normal meninges (*n*=31). Hierarchical clustering analysis showed distinct proteomic profiles of mutational subgroups revealing clusters of differentially expressed proteins (DEPs). Comparative analysis showed 10 proteins were commonly significantly upregulated (log2 fold-change≥1; *p*<0.05) among all mutational subtypes *vs*. normal meninges, indicating proteomic landscapes of mutational subtypes to be highly variable. In contrast, 257 proteins were commonly significantly downregulated (log2 fold-change≤-1; *p*<0.05) and enriched with molecular functions including aldehyde dehydrogenase and oxido-reductase. Mutational subtype-specific analysis identified 162 proteins significantly upregulated in *AKT1*^*E17K*^*/TRAF7 vs*. remaining sample groups to be enriched in the oxidative phosphorylation pathway. Lastly, analyses of 6600 phospho-sites (*n*=8) predicted regulatory kinases including EGFR and PKCα. Several of these up-regulated proteins and kinases already verified via WB. Further validation and functional verification will allow us to identify potential drug targets/biomarkers for meningioma.

